# Temperature dependence of nanoscale dynamic processes measured in living millipedes by high resolution inelastic and elastic neutron scattering

**DOI:** 10.1038/s41598-019-48270-6

**Published:** 2019-08-12

**Authors:** Eugene Mamontov, Naresh C. Osti, Madhusudan Tyagi

**Affiliations:** 1 Neutron Scattering Division, Oak Ridge National Laboratory, Oak Ridge, Tennessee 37831 USA; 2 NIST Center for Neutron Research and University of Maryland, Gaithersburg, Maryland 20899 USA

**Keywords:** Nanoscale biophysics, Multicellular systems

## Abstract

We have used high energy-resolution neutron scattering to probe nanoscale dynamic processes in living millipedes (*Narceus americanus*). We have measured the temperature dependence of the intensity of scattered neutrons that do not exchange energy with the living samples on the 1.5 ns time scale, thereby excluding the signal from the highly mobile intra- and extra-cellular bulk-like aqueous constituents in the sample. This measured “elastic” scattering intensity exhibits a non-monotonic temperature dependence, with a noticeable systematic decrease detected between 295 and 303 K on warming up from 283 to 310 K. This decrease demonstrates an excellent inverse correlation with the non-monotonic, as a function of temperature, increase in the slow diffusivity previously observed in planarian flatworms and housefly larvae. This correlation suggests the existence of a biological mechanism, possibly common between different classes (Insects and Myriapods) and even phyla (Arthropods and Platyhelminthes), that dampens the slow nanoscopic dynamics in ectothermic organisms in response to the temperature of the environment exceeding the physiologically optimal range.

## Introduction

**Figure 1:**
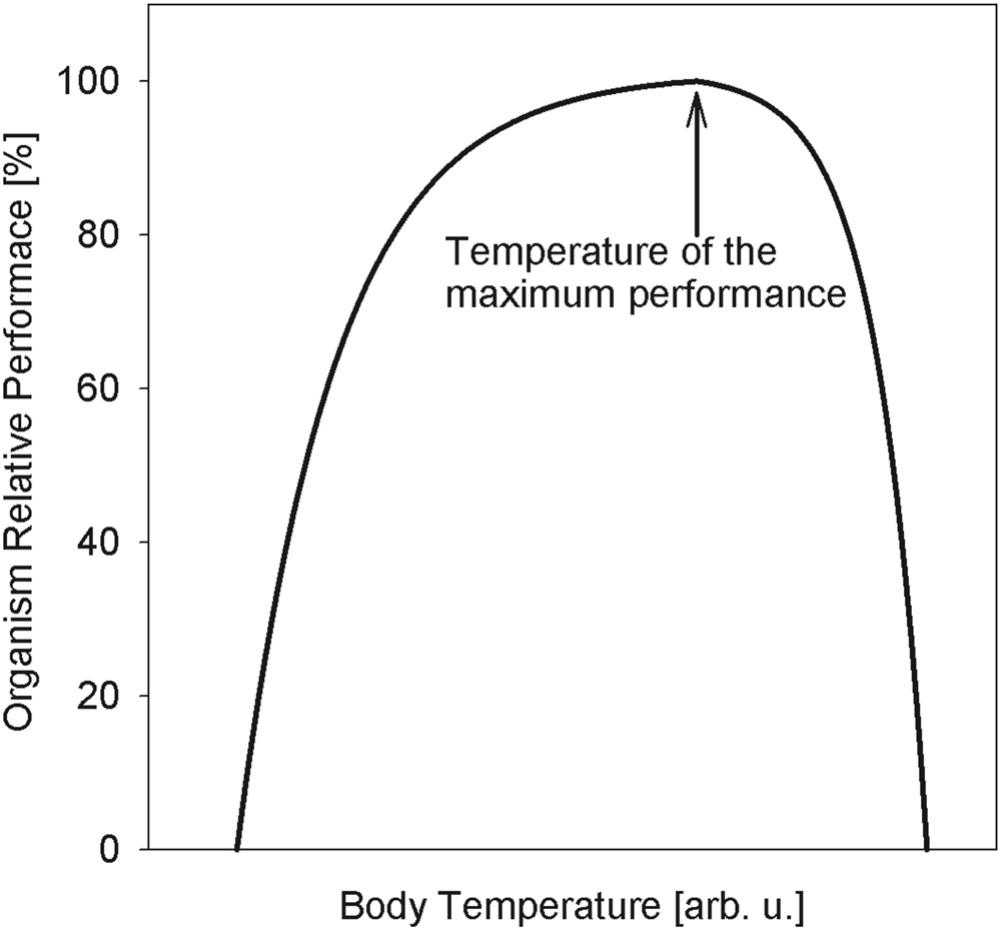
Schematic performance curve of an ectothermic organism as a function of temperature (as described in^1^).

Ectothermic animals are critically dependent on their environment to sustain the body temperature within the physiological range. Its boundaries are usually evident from the animal’s performance curve, as presented schematically in Fig. 1. The performance curve is an asymmetric function with respect to its maximum, with the range between the critical thermal minimum and the maximum performance temperature exceeding the range between the maximum performance temperature and the critical thermal maximum^1^. The fact that the optimal temperature of the maximum performance does not coincide with the critical thermal maximum shows that the performance does not merely increase as a function of temperature according to the Boltzmann’s factor, exp(−E_a_/k_B_T), all the way up until the organism can become damaged by high temperature^2^. Instead, the decrease in the performance beyond the optimal temperature due to the biological thermal stress factors^2^, which override the thermal activation-driven metabolic rate increase, implies the existence of physiological mechanisms responsible for slowing down the rate of biochemical metabolic reactions, which would not be expected from purely thermal activation standpoint.

In search of possible microscopic mechanisms underpinning the whole-body response of ectotherms to changing environment temperature, we have turned to high energy-resolution quasielastic neutron scattering measurements^3,4^. Unlike x-rays or electrons, cold or thermal neutrons primarily interact with samples via scattering (by nuclei) rather than direct ionization, except for the secondary prompt or delayed emission of ionizing particles following possible neutron capture. Irrespective of the relatively low neutron flux available at research reactors or accelerator-based sources, neutrons are a fundamentally weak probe with a low scattering and absorption cross-sections, and only a small fraction of nuclei in a sample interacts with neutrons in the course of a typical scattering experiment. In addition, energy resolution on the order of 10^−6^ eV is readily attainable in quasielastic neutron scattering experiments, providing the opportunity to probe dynamic processes on a nanosecond time scale. Finally, the dependence of scattering signal on the scattering momentum transfer reveals the geometry of molecular motions. While neutron scattering experiments on living animals have been attempted only recently^3,4^, less challenging systems, but still of considerable complexity, such as cell cultures^5–19^, tissues^20–22^, and brine shrimp eggs in the state of arrested metabolism^23,24^ have been more commonly probed using neutrons. In general, neutron scattering signal from biological samples in the native state is dominated by the hydrogen atoms due to their large incoherent scattering cross-section.

Compared to planarian flatworms of Platyhelminthes phylum^3^, insects of Arthropods phylum, such as housefly larvae^4^, exhibit higher morphological complexity, in particular, due to the presence of open circulatory system and hemolymph, yet both flatworms and larvae are soft-bodied animals. Here we study an organism of still higher complexity, a *Narceus americanus* millipede. Besides soft tissues, this myriapod features an extensive chitinous exoskeleton. Because stiff chitin much differs in its dynamic response from soft tissues, we find that its presence introduces an additional (elastic) component to the scattering signal and necessitates modification to the data collection and analysis procedure. Nevertheless, we show that the temperature dependence of the elastic scattering from millipede exhibits a remarkable inverse correlation with the non-monotonic temperature dependence of the slow diffusivity previously observed in planarian flatworms and housefly larvae, suggesting a universal mechanism at work that can dampen the microscopic dynamics in ectotherms in response to a rising temperature of the environment. To this end, we observe a remarkable analogy in the temperature dependence of the nanoscopic dynamics between the organisms representing different classes and phyla.

## Results and Discussion

**Figure 2:**
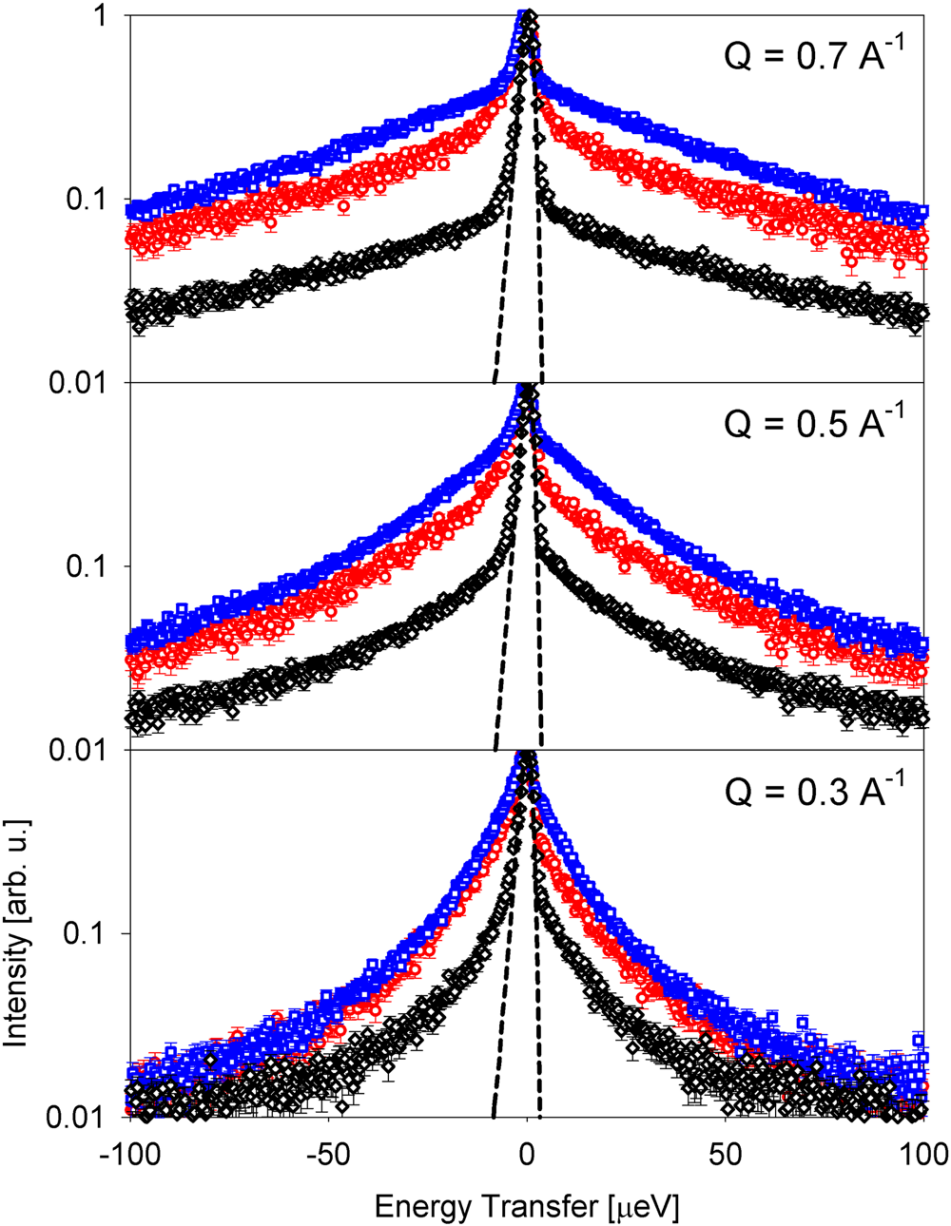
Scattering intensities I(Q, E) (background subtracted) from planarian flatworms at 290.7 K^3^ (red symbols), housefly larvae at 289.3 K^4^ (blue symbols) and millipede sample at 290.0 K (black symbols). The corresponding resolution measured from the millipede sample at 10 K is shown as the black dashed line. All spectra are normalized to unity. Error bars in all figures represent one standard deviation.

**Figure 3:**
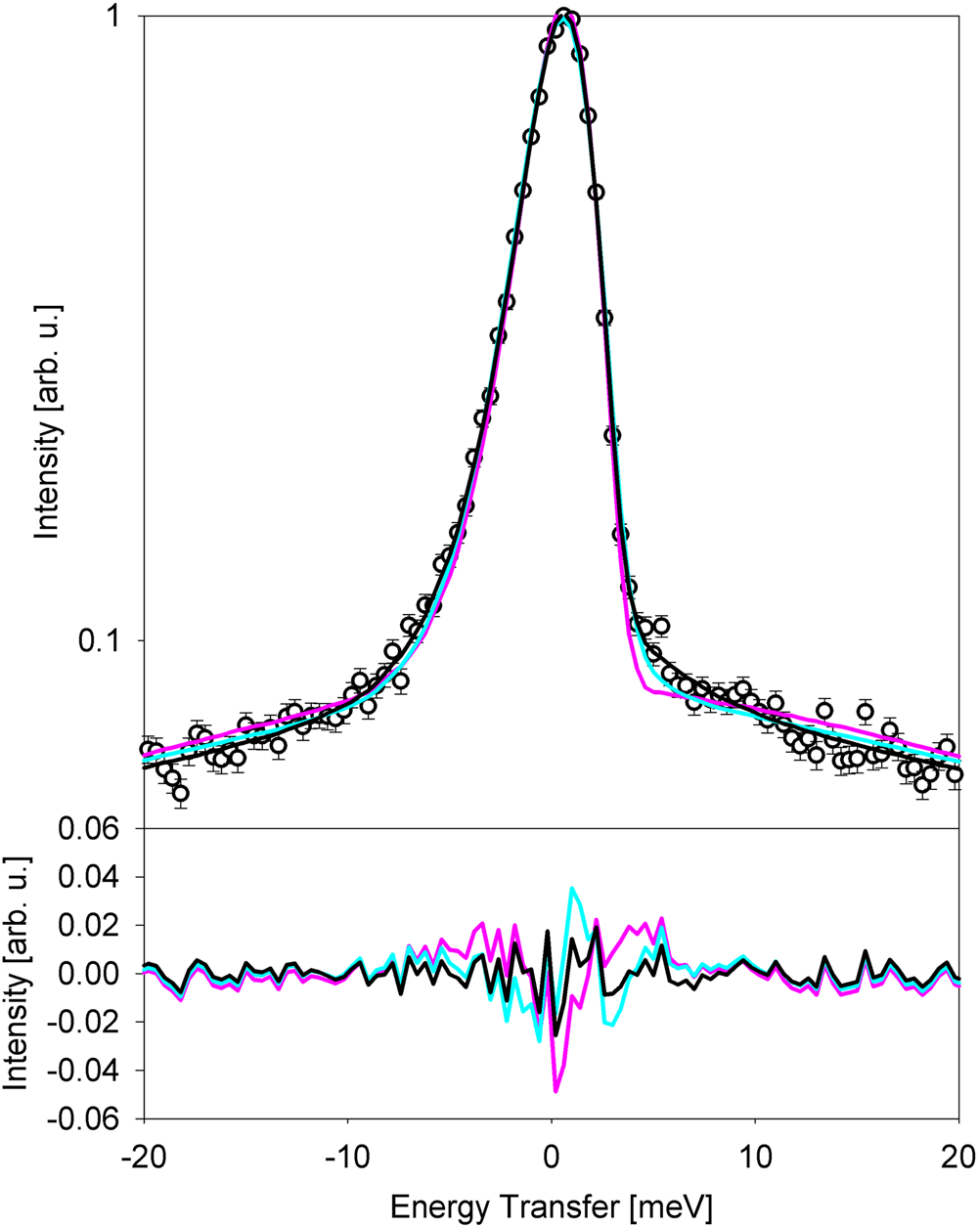
Comparison of fits obtained using different model scattering functions for millipede sample data at 290 K, Q = 0.7 Å^−1^ (symbols). The energy transfer range is truncated for better visibility, and the difference plots (data minus model) are shown in the bottom. Cyan lines: two Lorentzian functions (Eq. 1). Pink lines: a delta function plus a Lorentzian function (Eq. 2). Black lines: a delta function plus two Lorentzian functions (Eq. 3).

Figure 2 shows a comparison of the scattering intensities from different species (normalized to unity) measured on BASIS (see Materials and Methods). It is evident that, compared to the soft-bodied animals, the scattering signal from millipede sample exhibits a relatively stronger narrow component centered near zero energy transfer. This component appears almost, but not quite, as narrow as the elastic signal from the corresponding resolution function. As the chitin, which is abundant in millipedes, is rigid and thus expected to give rise to a purely elastic signal on the energy/time scale of a high energy-resolution quasielastic experiment, this shape of the scattering signal from millipede sample is rather intuitive. On the other hand, it is not obvious that the fit models used for the soft-bodied animals^3,4^ will remain adequate for the description of quasielastic signal from millipedes. To this end, we applied various fit models to the data, as shown in Fig. 3.

The first fit model, successfully used in the past for soft-bodied planarian flatworms and housefly larvae^3,4^, features a superposition of two Lorentzian functions, narrow and broad, convolved with the resolution function, R(Q,E), and a linear background term:

1 $$\begin{array}{c}I(Q,E)=[x(Q)\frac{1}{\pi }\frac{{{\rm{\Gamma }}}_{n}(Q)}{{{\rm{\Gamma }}}_{n}{({\rm{Q}})}^{2}+{E}^{2}}+(1-x(Q))\frac{1}{\pi }\frac{{{\rm{\Gamma }}}_{b}({\rm{Q}})}{{{\rm{\Gamma }}}_{b}{({\rm{Q}})}^{2}+{E}^{2}}]\otimes R(Q,E)\\ \,\,\,\,+({C}_{1}(Q)E+{C}_{2}(Q))\end{array}$$

For the data presented in Fig. 3, this model gives an agreement factor, defined as *χ*^2^ = Σ(*I*_experiment_ − *I*_model_)^2^/(σ^2^ (*N*_observations_ − *N*_parameters_)), where σ^2^ is the variance, of 1.256, yet the zoomed-in difference plot in Fig. 3 reveals a systematic discrepancy between the fit and data near the central peak. Evidently, the narrow Lorentzian component does not describe well the resolution-defined elastic scattering intensity, which therefore needs to be accounted for explicitly. Therefore, our next fit model featured a delta-function and a Lorentzian function:

2 $$I(Q,E)=[x(Q)\delta (E)+(1-x(Q))\frac{1}{\pi }\frac{{\rm{\Gamma }}(Q)}{{{\rm{\Gamma }}(Q)}^{2}+{E}^{2}}]\otimes R(Q,E)+({C}_{1}(Q)E+{C}_{2}(Q))$$

When convolved with the resolution function, the delta function gives rise to the resolution-defined elastic scattering signal. However, using this model, we obtained even worse agreement factor, *χ*^2^ = 1.557, and no improvement in the systematic discrepancy between the fit and data near the elastic peak. Finally, applying a fit model featuring a delta-function and two Lorentzian terms,

3 $$\begin{array}{c}I(Q,E)=[x(Q)\delta (E)+(1-x(Q))(y(Q)\frac{1}{\pi }\frac{{{\rm{\Gamma }}}_{n}({\rm{Q}})}{{{\rm{\Gamma }}}_{n}{({\rm{Q}})}^{2}+{E}^{2}}+(1-y(Q))\frac{1}{\pi }\frac{{{\rm{\Gamma }}}_{b}({\rm{Q}})}{{{\rm{\Gamma }}}_{b}{({\rm{Q}})}^{2}+{E}^{2}})]\,\otimes \\ \,\,\,\,\,\,R(Q,E)+({C}_{1}(Q)E+{C}_{2}(Q))\end{array}$$

we obtained an excellent agreement, *χ*^2^ = 1.072, and no systematic discrepancy between the fit and data near the elastic peak, as one can see in Fig. 3 difference plot.

**Figure 4:**
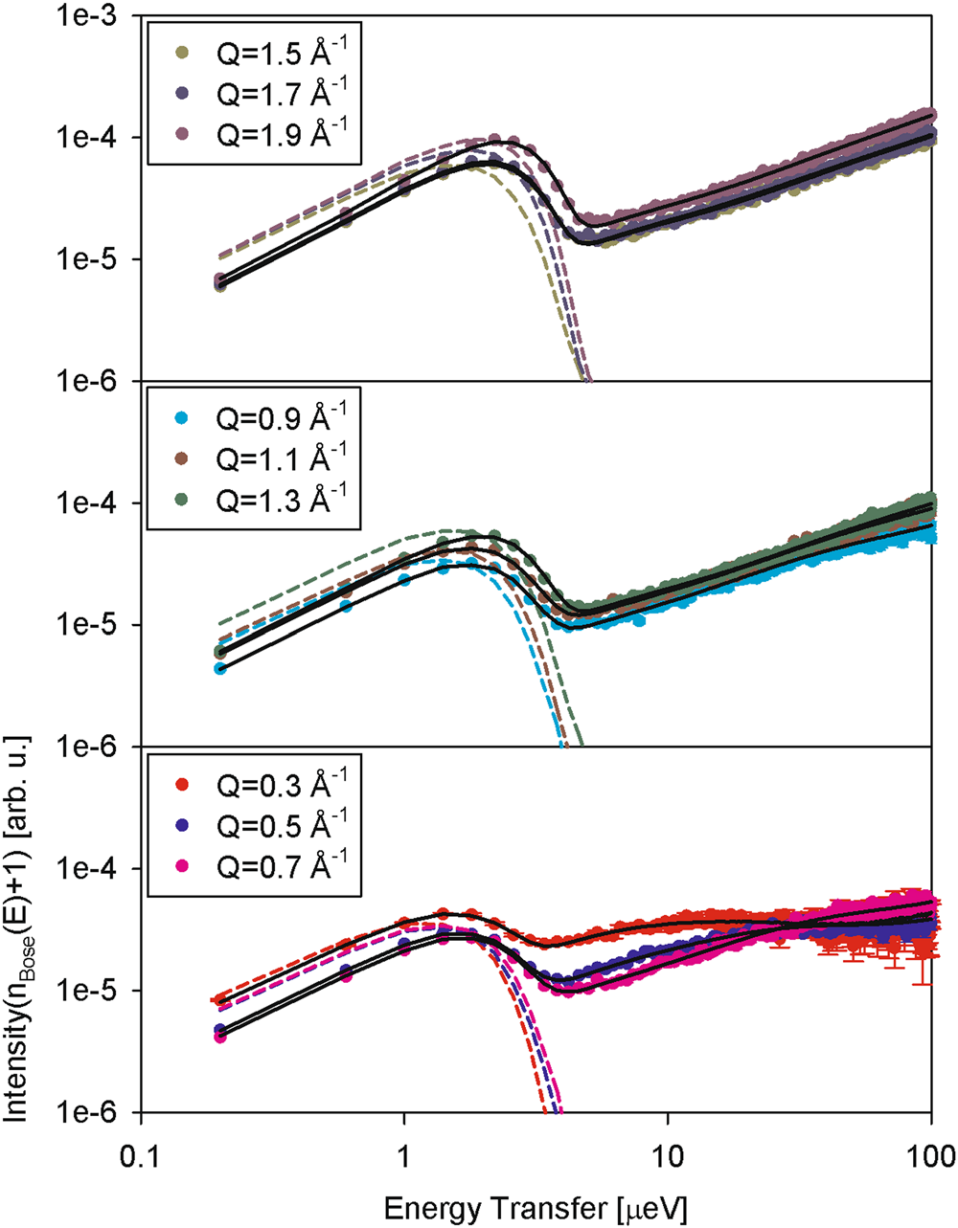
The log-log plot of the neutron scattering signals from millipede sample at 290 K divided by Bose population factor, I(Q, E)/(n_Bose_(E) + 1). Solid black lines show fits with Eq. 3. Dashed colored lines show the rescaled resolution function (Q-dependent).

**Figure 5:**
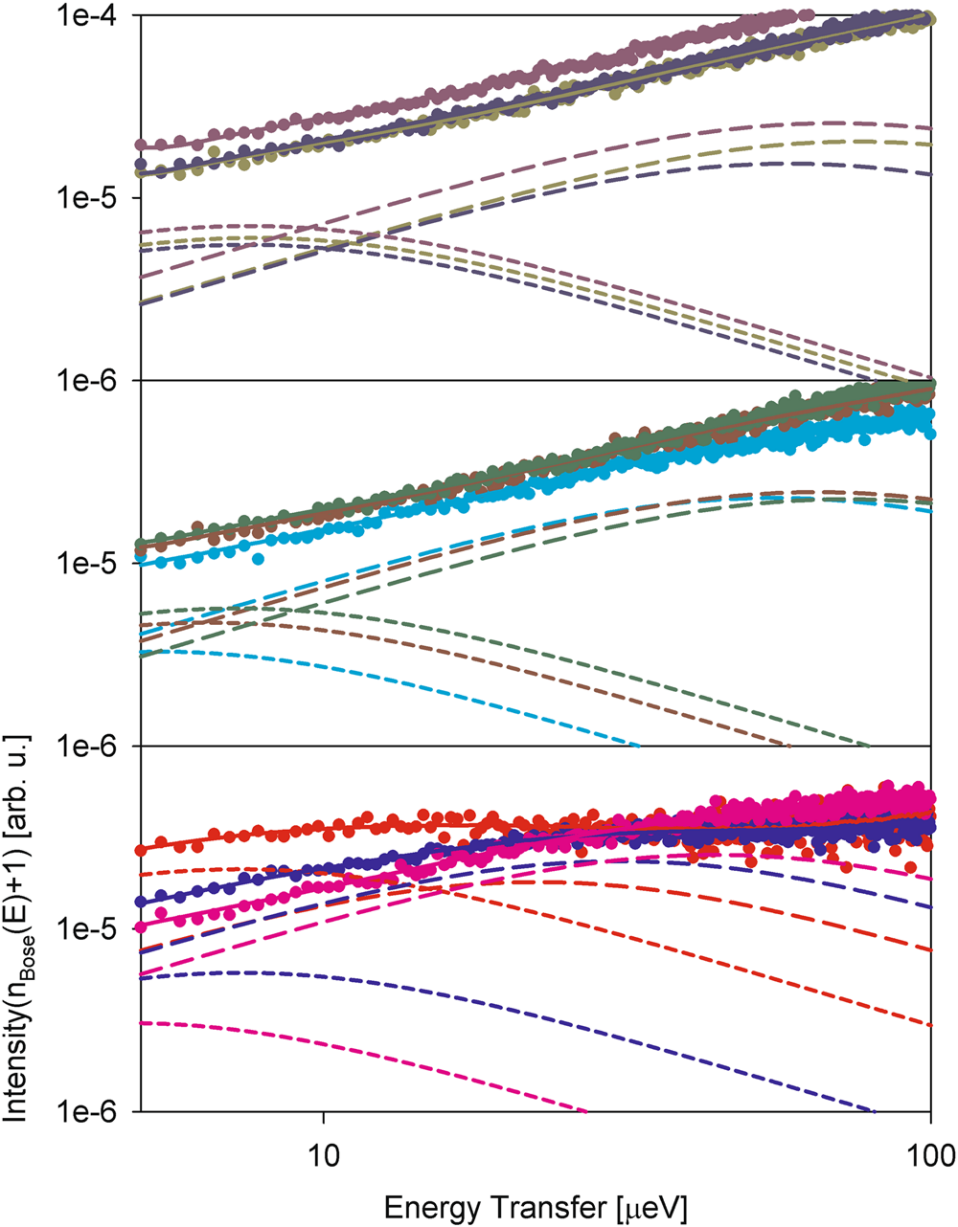
Symbols: the same neutron scattering signals as shown in Fig. 4, but with subtracted elastic line intensity (as fitted using Eq. 3). Solid lines show the overall fit of the signals obtained with Eq. 3, which includes a narrow component (short-dashed lines), a broad component (long-dashed line), and the background term (not shown).

While fit models described by Eqs 1–3 are empirical, it is also instructive to evaluate model-independent data. To this end, Fig. 4 shows the data displayed as I(Q, E)/(n_Bose_(E) + 1), where I(Q, E) is measured neutron scattering signal, n_Bose_(E) = (exp(E/k_B_T) − 1)^−1^ is Bose population factor, and k_B_ is Boltzmann’s constant. At higher energy transfers, where the influence of the spectrometer resolution is relatively weak, such data presentation approximates the imaginary part of the dynamic susceptibility, χ”(Q, E). The maxima of dynamic susceptibility correspond to the characteristic relaxation frequencies in the system, thus enabling model-free, albeit qualitative, data visualization and analysis. While the data in Figs 2 and 3 demonstrate the presence of the elastic (on the experiment time scale) component in the scattering signal from the millipedes, Fig. 4 emphasizes that the non-elastic scattering signal comprises at least two distinct dynamics components, as was previously observed for the planarian flatworms^3^ and housefly larvae^4^. This is further illustrated by Fig. 5, which shows the same data as in Fig. 4, but with subtracted elastic line intensity. The data in Fig. 5 are not completely model-independent, as the exact elastic intensity to be subtracted had to be determined by fitting I(Q, E) with Eq. 3 to obtain the x(Q) parameter, yet they provide the best presentation of the QENS data without the elastic scattering contribution. The broad signals presented in Fig. 5 provide further evidence that the non-elastic scattering in the I(Q, E) data (without the elastic line contribution) could not be described by a single Lorentzian component, even with a background, and had to include a narrower and a broader component, as shown by the dashed lines in Fig. 5.

**Figure 6:**
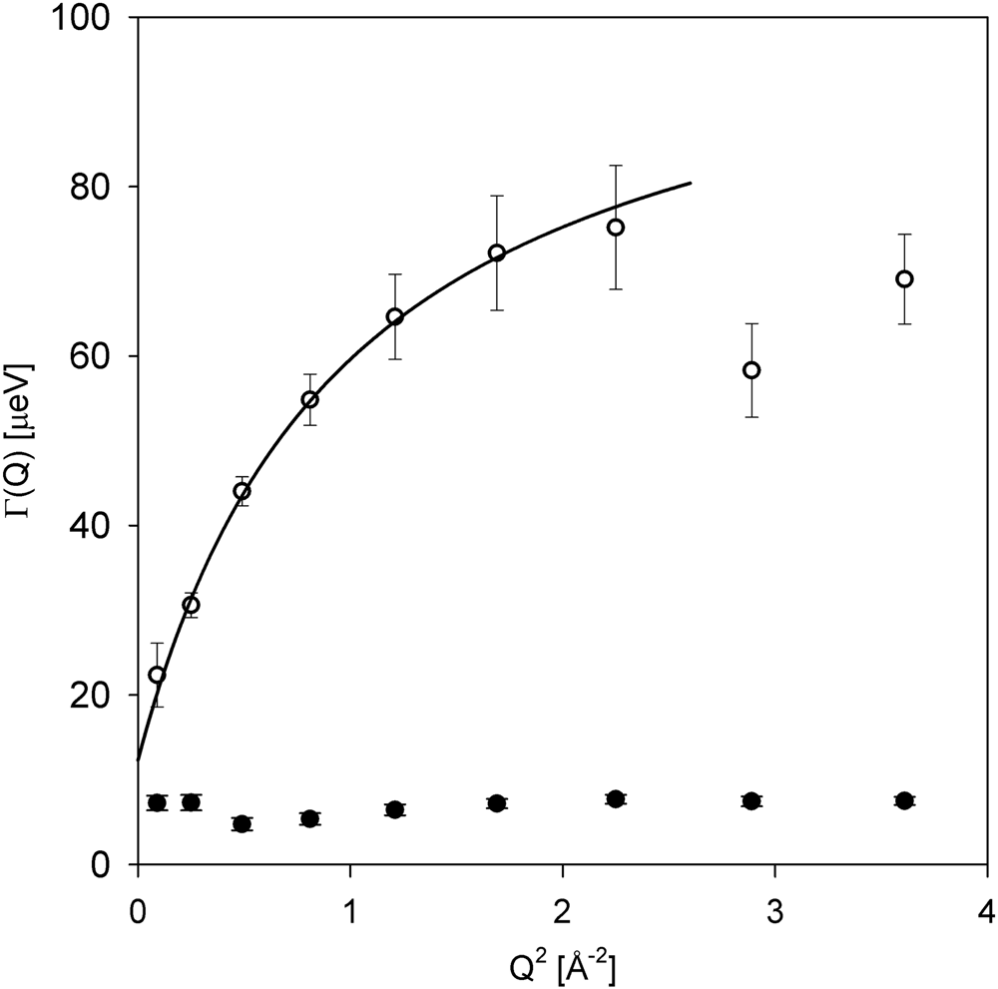
Width (HWHM) of broad (open symbols) and narrow (filled symbols) dynamic components obtained from a fit of the millipede sample data at 290 K using a delta function and two Lorentzian functions (Eq. 3). Solid line: fit (up to Q = 1.5 Å^−1^) of the broad dynamic component with a jump diffusion model described by Eq. 4.

The broader component has been previously linked^3,4^ to the bulk-like aqueous constituents, such as intracellular water or hemolymph. Likewise, the Q dependence of the higher-E data presented in Fig. 4 suggests that the signal from the millipedes in the higher energy transfer region is dominated by scattering from the bulk-like aqueous constituents in the sample. Indeed, when the Q-dependence of the broad Γ_b_(Q) component obtained from fit with Eq. 3 is fitted, as displayed in Fig. 6, with a modified jump diffusion model,

4 $${{\rm{\Gamma }}}_{b}(Q)=B+\frac{\hslash D{Q}^{2}}{1+\tau D{Q}^{2}}$$

we obtain the diffusivity (D) = (14.5 ± 4.6) × 10^−10^ m^2^/s, the residence time between jumps (τ) = (7.0 ± 1.0) ps, and the offset parameter (B) = (12.3 ± 4.8) μeV. The fits are performed to a maximum Q of 1.5 Å^−1^; beyond that range, the strong coherent scattering at the structural maximum position starts interfering with the otherwise predominantly incoherent scattering signal by the hydrogen atoms in the sample. The diffusivity and residence time values for bulk water at 290 K are ca. 19 × 10^−10^ m^2^/s and 1 ps, respectively^25,26^. The broad component-derived value of D obtained for the millipede sample is between those measured for housefly larvae^4^ and planarian flatworms^3^, and agrees with the general notion^5,10,12,16^ that the diffusivity of cytoplasmic water is comparable with bulk water diffusivity, even though the residence time between diffusion jumps in the former could be longer by an order of magnitude. The offset parameter B is employed to account for effects due to multiple scattering of neutrons in the sample when the sample thickness renders them unavoidable^3,4,27^, and is implicitly taken as zero in standard fit models when the sample thickness can be controlled. Therefore, this offset parameter may be expected to grow with the increasing contribution of multiple scattering effects. Indeed, in the experiments on flatworm planarians and housefly larvae, with a mass of less than 1 g for each sample, this parameter for the aqueous component was^3,4^ about 4 μeV, whereas it has increased to ca. 12 μeV in the current measurement of the 7.555 g millipede sample, indicating more pronounced multiple scattering effects in the sample.

From the analysis described above, it can be concluded that a fit model with a delta function (accounting for the elastic scattering contribution, e.g., from the chitin) and two Lorentzian functions, the broader of which originates from the bulk-like aqueous constituents of the sample, would be the most appropriate. However, the presence of elastic component would complicate quantitative analysis of temperature-dependent data. It is not obvious that the temperature evolution of the width of the narrow Lorentzian component would be directly correlated with the diffusivity, as was the case for the soft-bodied animals with no elastic scattering signal. Redistribution of the scattering intensity between the elastic and narrow Lorentzian components, instead of, or together with, change in the narrow Lorentzian component width, could be taking place as a function of temperature. In view of this complication, we pursued an alternative method to monitor the evolution with temperature of the nanoscopic dynamics in millipedes, using temperature scans of the elastic intensity. While time-of-flight backscattering spectrometers, such as BASIS, excel in delivering a broad range of accessible energy transfers and can collect temperature scans of the elastic intensity too^28^, the latter mode of operation is much more efficient on reactor-based backscattering spectrometers^28^, such as HFBS (see Materials and Methods). With the energy resolution of HFBS of 0.8 μeV (FWHM), dynamic processes slower than ca. 1.5 ns on the nanometer length scale (defined by the Q range of the spectrometer) will contribute to the elastic scattering intensity.

**Figure 7:**
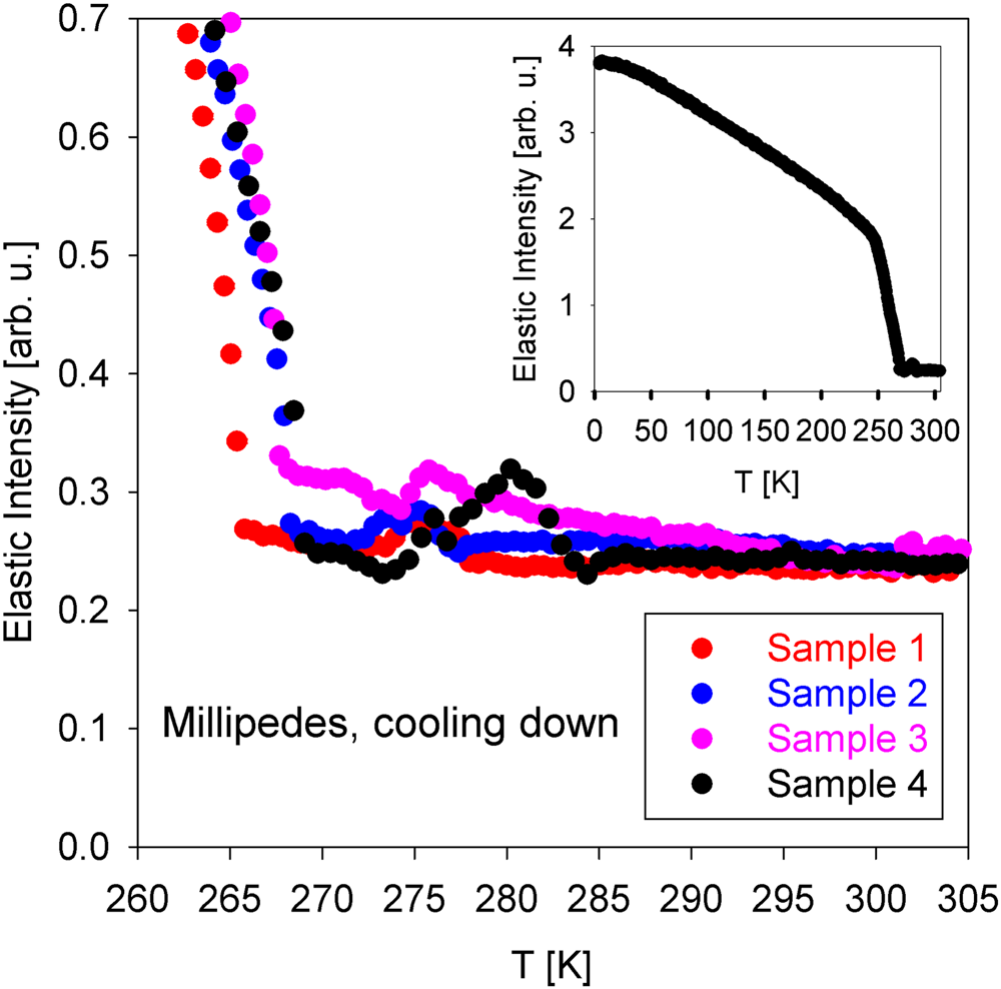
Temperature dependence of the elastic scattering intensity (normalized to the specimens mass) from millipede samples on cooling down at 1 K/min. Inset: full temperature range (measured with Sample 4 only). The data were summed up for 0.25 Å^−1^ < Q < 1.75 Å^−1^.

Figure 7 shows the temperature dependence of the elastic scattering intensity from several millipede samples collected on cooling down. Although the relatively fast temperature scan rate, necessary to cover a broad temperature range, precluded proper temperature equilibration at measurement points, these diagnostic elastic intensity scans were useful for observation of the freezing pattern in millipedes. Major freezing begins below ca. 268 K, as one can see from the main panel, and becomes complete by the time the temperature reaches 250 K, as evident from the inset. Interestingly, there is evidence of some apparently non-aqueous constituents freeing, which varies among the samples, but generally takes place between 283 and 273 K. Furthermore, the elastic scattering intensities at ambient temperatures are rather similar among four samples after normalization to the sample mass, despite no special alignment for individual samples, as can be expected for the beam size of 30 mm by 30 mm that exceeds the dimensions of the coiled millipede samples.

**Figure 8:**
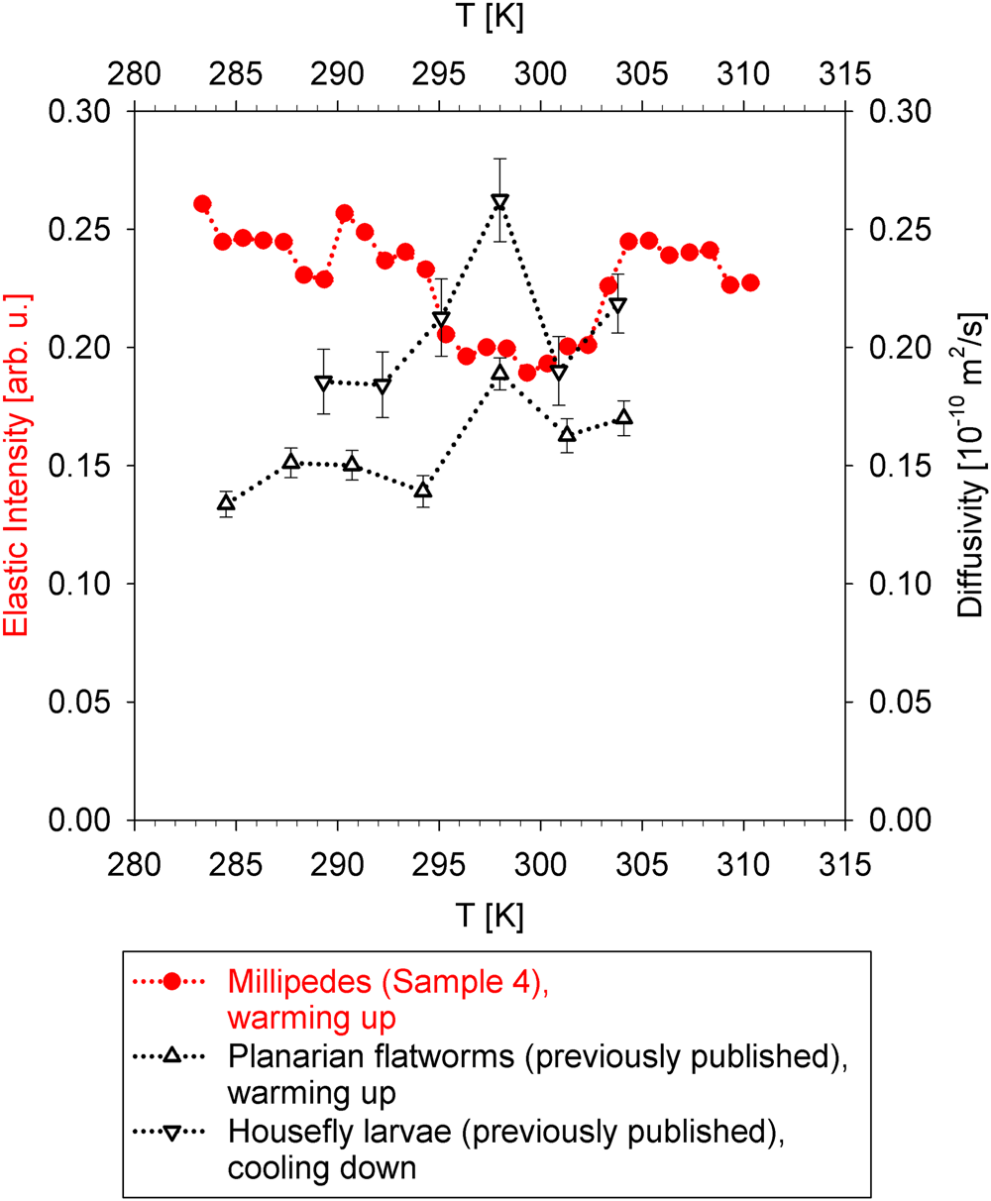
Red filled circles: temperature dependence of the elastic scattering intensity (values shown on the left axis) from millipede sample on warming up at 0.2 K/min. Error bars are within the symbols. Open up-triangles: microscopic diffusivity (values shown on the right axis) measured for planarian flatworms on warming up^3^. Open down-triangles: microscopic diffusivity measured for housefly larvae on cooling down^4^. Dotted lines are guide for the eye. The elastic scattering intensity data were summed up for 0.25 Å^−1^ < Q < 1.75 Å^−1^.

Before Sample 4 was frozen, its elastic scattering intensity was carefully measured on warming up, as shown in Fig. 8. The collection of the entire warming up data set of elastic scattering intensity took less than 2.5 hours, which approximately equals the collection time of the full dynamic data set at one temperature in the BASIS measurement, thus illustrating the effectiveness of such approach on the HFBS. Furthermore, the slow temperature scan rate and the long-time equilibration of the sample environment equipment at 283 K prior to the warming up scan allowed for the proper temperature equilibration and accurate measurements of the sample scattering response.

Despite some scatter in the data, there is a noticeable reversible systematic decrease in the elastic scattering intensity visible between 295 and 303 K in Fig. 8. While the effects due to multiple scattering in the sample influence the counts, they are not expected to change reversibly between 295 and 303 K. As the contribution from the broad, bulk-like aqueous constituents-related dynamic component to the elastic scattering intensity is very weak in the investigated temperature range of 283 to 310 K, the data in Fig. 8 represent a contribution from both the elastically scattering constituents and the narrow, slow dynamic component in the sample. Likewise, the dynamic components that are too broad for the BASIS accessible energy transfer range have even smaller influence on the temperature dependence of the elastic intensity presented in Fig. 8.

The intensity dip observed between 295 and 303 K is indicative of an increase in the softness in the millipede sample in this temperature region. Remarkably, this decrease shows a correlation with the temperature range of the enhanced diffusivity of the slow-moving constituents in the previously measured planarian flatworms^3^ and housefly larvae^4^, which are also presented in Fig. 8. The non-monotonic temperature dependence of the diffusivity/softness is reminiscent of the schematic performance curve presented in Fig. 1.

Assignment of the non-monotonic with temperature nanoscopic dynamics to the specific constituents has been challenging even for the less morphologically complex organisms^3,4^. Lipid assemblies, present in the organisms in considerable quantitates^29–33^, have been suggested as possible contributors to the non-monotonic temperature dependence. However, it should be noted that both the global protein diffusivity and the dynamics of “hydration” water^16^ in contact with the cellular constituents also exhibit prominent contribution to the scattering signal in the μeV energy transfer range. Unambiguous assignment of the slow dynamic component, which strongly influences the elastic scattering intensity, may be challenging or impossible in systems of such high complexity as living organisms. Nevertheless, irrespective of the specific assignment of the nanoscopic dynamics, the data presented in Fig. 8 provide additional support to the idea of the universal, non-monotonic temperature dependence of the nanosecond time scale dynamics underpinning the whole-body animal performance curve. While some results published to date show a non-trivial temperature dependence of the nanoscopic dynamics of hemoglobin, varying among different species of animals^8,34–37^, apparently as an evolutionary adaptation of organisms, our observations reveal some universal biophysical factors, given the substantial difference not only in the phyla classification, but also in the living environment conditions, between planarian flatworms, housefly larvae, and millipedes.

In conclusion, quasielastic neutron scattering measurements of nanoscopic dynamics in living millipede samples have revealed a broader, water-like dynamic component from the bulk-like aqueous constituents, a narrower dynamic component, and an elastic component, likely associated primarily with the chitinous exoskeleton. Thus, the quasielastic neutron scattering spectrum from millipedes is considerably more complex compared to those previously obtained from soft-bodied animals. The temperature-dependent intensity of scattered neutrons that do not exchange energy with the millipede samples on the 1.5 ns timescale, thereby excluding the signal from the highly mobile intra- and extra-cellular bulk-like aqueous constituents in the sample, has been analyzed. When measured on warming up from 283 to 310 K, this “elastic” scattering intensity exhibits a dip between 295 and 303 K. This increase in the softness of millipede sample demonstrates a remarkable correlation with the temperature range of the enhanced diffusivity of the slow-moving constituents in the previously measured planarian flatworms and housefly larvae. Given the substantial difference among these organisms, not only in the classification, but also the living environment conditions, our observations suggest the existence of a universal biological mechanism that overrides the thermal activation-driven metabolic rate increase and dampens the nanoscopic dynamics in ectotherms in response to a rising temperature of the environment before the organism can be damaged. This nanoscopic-level mechanism must be underpinning the whole-body performance curve observed in ectothermic animals.

## Materials and Methods

Living specimens of *Narceus americanus* millipedes were purchased from Carolina Biological Supply Co. A specimen used in the measurement at BASIS neutron spectrometer weighed 7.555 g. Specimens 1, 2, 3, and 4 used in the measurements at HFBS neutron spectrometer weighed 7.852, 5.566, 12.350, and 3.863 g, respectively. Immediately prior to the measurement, each specimen was loaded and sealed in a cylindrical aluminum container of 54 mm height and 29 mm inner diameter. Survival of millipedes in sealed containers at both ambient temperature and 310 K for at least 2.5 hours was verified using specimens not utilized in the scattering experiments. The temperature of the containers with the specimens was controlled during the neutron scattering experiments within ±0.2 K using a closed-cycle refrigerator in the standard operation mode, with helium thermal exchange gas surrounding the sealed containers.

A neutron backscattering spectrometer BASIS^38^ at the Spallation Neutron Source, Oak Ridge National Laboratory, provided a range of neutron energy transfers suitable for data analysis between −100 µeV and +100 µeV, with the energy resolution (averaged over all scattering angles) of 3.8 µeV (full width at half maximum). Routine data reduction procedures, including background subtraction and normalization to vanadium standard, were utilized^39^. The measurements at 290 K for 2 hours were followed by fast cooling down to 10 K and collecting the sample-specific resolution function, also for 2 hours. A neutron backscattering spectrometer HFBS^40^ at the NIST Center for Neutron Research was operated in a fixed-window mode, monitoring the intensity of neutrons scattered by the sample “elastically”, that is, with the energy exchange not exceeding 0.8 µeV (full width at half maximum of the energy resolution, FWHM), as a function of temperature. The temperature scans of all samples were concluded below the freezing temperature of the specimens (only cooling down was used for Sample 1, 2, and 3, whereas warming up followed by cooling down was used for Sample 4). The temperature ramp rate was 1 K/min on cooling down and 0.2 K/min on warming up. Besides, the temperature of the sample environment equipment was equilibrated for half an hour before installation of the sample for the measurement on warming up.

## Data Availability

The datasets analyzed during the current study are available from the corresponding author on reasonable request.
